# Overall leukocyte levels link risk factors to Von Willebrand factor and Neutrophil Extracellular Traps in stroke thrombi: a Structural Equation Modeling analysis

**DOI:** 10.3389/fneur.2025.1515596

**Published:** 2025-06-23

**Authors:** Lin Shi, Chunxiao Wei, Guowei Ye, Panpan Zhao, Shiqiang Ma, Yiming Qi, Ruolin Zhou, Yaru Zhang, Yan Zhang, Lingjie Meng, Weihang Xu, Chao Li, Shouchun Wang, Li Sun

**Affiliations:** ^1^Department of Neurology and Neuroscience Center, The First Hospital of Jilin University, Jilin University, Changchun, China; ^2^Department of Intensive Care Unit, The First Hospital of Jilin University, Changchun, China; ^3^Department of Neurosurgery Center, The First Hospital of Jilin University, Changchun, China; ^4^EEG Laboratory, Department of Neurology, Wei Fang Shi Ren Min Yi Yuan, Weifang, Shandong, China; ^5^Department of Neurosurgery, Huashan Hospital, Fudan University, Shanghai, China; ^6^School of Public Health, Jilin University, Changchun, China

**Keywords:** stroke, overall leukocyte levels, Von Willebrand factor (VWF), Neutrophil Extracellular Traps (NETs), Partial Least Squares Structural Equation Modeling (PLS-SEM)

## Abstract

**Background:**

Von Willebrand Factor (VWF) and Neutrophil Extracellular Traps (NETs) are involved in the inflammatory response during thrombi formation and are widely found in thrombi of Acute Ischemic Stroke (AIS) patients. Inflammation may mediate the relationship between cerebrovascular risk factors (such as blood glucose) and thrombi components. This study uses overall leukocyte levels to identify potential links between risk factors and VWF, NETs in thrombi.

**Methods:**

Thrombi samples and clinical data from 61 stroke patients treated at our hospital between 2017 and 2023 were collected. The Partial Least Squares Structural Equation Modeling (PLS-SEM) assessed direct and indirect associations, with leukocyte and its subtype counts as mediating variables, VWF and NETs as endogenous variables, and cerebrovascular risk factors as exogenous variables.

**Results:**

Heart dysfunction and blood glucose showed a significant negative indirect effect on VWF through the overall leukocyte levels (indirect effect = −0.119 and −0.118, *p* < 0.05). Overall leukocyte levels acted as a mediator between vitamin B12 and VWF levels in thrombi, displaying a positive mediatory impact (indirect effect = 0.118, *p* < 0.05). The decrease in VWF levels within thrombi was closely associated with an increase in discharge NIHSS scores (path coefficient = −0.353, *p* < 0.05). Additionally, overall leukocyte levels and homocysteine (Hcy) had significant negative effects on VWF (path coefficients = −0.384 and −0.308, *p* < 0.05), while vitamin B12 had a negative impact on NETs (path coefficient = −0.289, p < 0.05).

**Conclusion:**

Overall leukocyte levels mediate the influence of blood glucose levels, heart dysfunction, and vitamin B12 on content VWF in thrombi in stroke patients. VWF reduction correlates with elevated discharge NIHSS scores. These cerebrovascular factors may regulate the pathological process of AIS thrombosis through inflammatory responses, guiding further exploration of the underlying mechanisms and informing clinical strategies.

## 1 Introduction

Stroke is one of the leading causes of death in China, with an incidence of approximately 505.2 per 100,000 person-years and a mortality rate of 343.4 per 100,000 person-years (1). The development of mechanical thrombectomy has significantly improved the treatment outcomes of Acute Ischemic Stroke (AIS) and has also facilitated research on thrombi components (2). Recent analyses of thrombi components have enhanced our understanding of various pathological processes involved in thrombogenesis, providing new insights for future therapies (3). Among these components, Von Willebrand Factor (VWF) and Neutrophil Extracellular Traps (NETs) are commonly found in thrombi samples.

VWF is large multimeric glycoprotein released by activated endothelial cells during stroke, mediating platelet adhesion to injured or activated vascular walls and contributing to thrombosis. VWF also mediates inflammatory processes in brain through the collagen–VWF–GPIb axis, promoting leukocyte adhesion, rolling, and extravasation (4). Inflammatory responses significantly enhance VWF activation (5). Studies have shown that targeting the interaction between platelet GPIbα and VWF can inhibit thromboinflammation, thereby protecting neural cells (6). NETs are extracellular web-like structures formed by neutrophils, consisting of decondensed chromatin associated with antimicrobial proteins and peptides (7). In AIS, NETs were initially discovered as a novel form of neutrophil-mediated immune process. NETs form fibrous structures with potent antimicrobial properties (8) and can activate the intrinsic coagulation cascade, shorten clotting time and accelerate thrombosis (9). As vital components of thrombi, NETs and VWF contribute to the inflammatory response during thrombi formation (4).

Studies reveal that the inflammation triggered by cerebral ischemia plays a key role in stroke exacerbation. This process is regulated by various risk factors, including lipids (10), blood glucose (11), homocysteine (Hcy) (12), vitamins B (13), liver function (14), kidney function (15, 16), and levels of sodium and calcium (17). Compared to traditional medical statistical methods, Structural Equation Modeling (SEM) is a powerful multivariate analysis technique that can validate multiple regression equations simultaneously. It enables the consolidation of multiple observed variables sharing analogous characteristics into latent variables. It elucidates the structural interrelationships among these constructs to yield a comprehensive, sophisticated model by estimating path coefficients. It supports the evaluation of complex direct and indirect causal relationships between dependent and independent variables (18). SEM has become widely utilized by clinical scientists as a data analysis tool in various fields (19–22). Partial Least Squares SEM (PLS-SEM) is suitable for models with multiple indicators, single measurements, small sample sizes, and relaxed distribution assumptions (23).

Our study uses PLS-SEM for data analysis, focusing on overall leukocyte levels as inflammatory marker, to examine whether cerebrovascular risk factors affect NETs and VWF content in thrombi through inflammatory responses.

## 2 Materials and methods

### 2.1 Sample collection

Thrombi were collected from AIS patients underwent mechanical thrombectomy in the emergency department of our hospital between 2017 and 2023. A total of 87 thrombi specimens were obtained, of which 26 were excluded due to insufficient clinical data. The procedures were performed entirely by specialized neurointerventional physicians, with guidance of experienced radiologists. The ethics committee approved the study and we obtained the verbal agreement of the patients. (As this study was retrospective and did not involve any diagnostic or therapeutic interaction with patients, all specimens and clinical data were collected during routine care. The research procedures posed no risk of disclosing personal information and did not subject patients to additional harm or discomfort.)

### 2.2 Clinical data

The collected clinical data included: age, sex, peripheral serum test results upon admission (4–6 h after onset): lipid levels (including serum cholesterol, triglycerides, low-density lipoprotein cholesterol, and high-density lipoprotein cholesterol); Hcy; vitamin B12; cardiac dysfunction markers (including creatine kinase-MB (CKMB), B-type natriuretic peptide (BNP) and myoglobin); overall leukocyte levels (absolute counts of neutrophils, lymphocytes and eosinophils); liver function parameters (aspartate aminotransferase (AST), alanine aminotransferase (ALT), alkaline phosphatase, *γ*-glutamyl transferase, and cholinesterase); bilirubin levels (total, direct, and indirect bilirubin); renal function indices (serum creatinine, urea nitrogen, and uric acid); serum protein levels (total protein, albumin); electrolyte levels (serum potassium, sodium, chloride, and calcium); blood glucose (random glucose, glycated hemoglobin, and fasting glucose). National Institutes of Health Stroke Scale (NIHSS) scores at admission and discharge.

### 2.3 Sample preparation

Thrombi retrieved were rinsed with saline, and immediately incubated at room temperature in 4% paraformaldehyde for 24 h. The samples were then embedded in paraffin and sectioned into 5 um slices. Immunohistochemical staining for VWF and NETs was performed by VWF (D8L8G) XP Rabbit mAb(#65707) (CST) and anti-Histone H3 (citrulline R2 + R8 + R17) antibody [RM1001] (ab281584) (Abcam).

### 2.4 Image analysis

Samples were photographed by OLYMPUS BX51 fluorescence microscope with a digital camera at 400x magnification. Five different fields of view were selected for each thrombi. ImagePro Plus software analyzed the acquired images for area measurement and data quantification. The levels of NETs and VWF in thrombi were quantified as average optical density (AOD), defined by the ratio of IOD value to the positive area.

### 2.5 Data analysis

R Studio 2023.6.0.421 and SmartPLS 4.0.9.6 were used to perform statistical analyses. The study applied PLS-SEM to construct models since all data were continuous variables with non-normal distributions (24). Laboratory indices with similar functions were combined as latent variables. To assess the impact of various indicators on NETs and VWF content in AIS thrombi and the mediating role of overall leukocyte levels, we first specified an initial PLS-SEM model including all observed and latent variables, then refined it iteratively based on each latent construct’s indicator loadings and overall model-fit criteria. Some data were standardized and transformed by Box-Cox transformation based on distribution characteristics. The samples are stratified by potential confounders (age, sex, and clinical history), tested intergroup differences using the Mann–Whitney U test.

## 3 Results

### 3.1 Data overview

**Figure 1:**
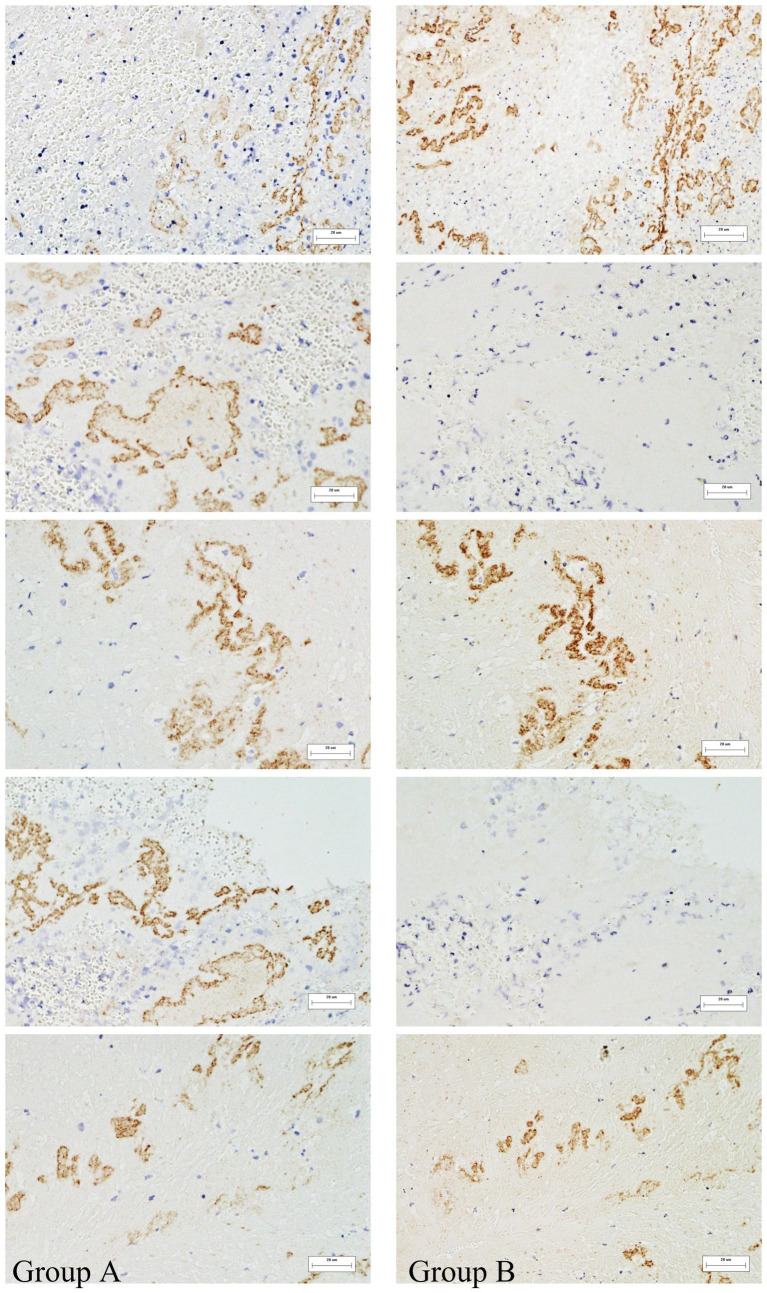
Immunohistochemical analysis of thrombi specimens. Representative images showing VWF and NETs staining in thrombi of different origins. These images illustrate the presence and spatial distribution of the two markers within thrombus sections. Immunohistochemically positive areas are shown in brownish-yellow. **(A)** Represents NETs, **(B)** represents VWF. 400x magnification.

**Figure 2:**
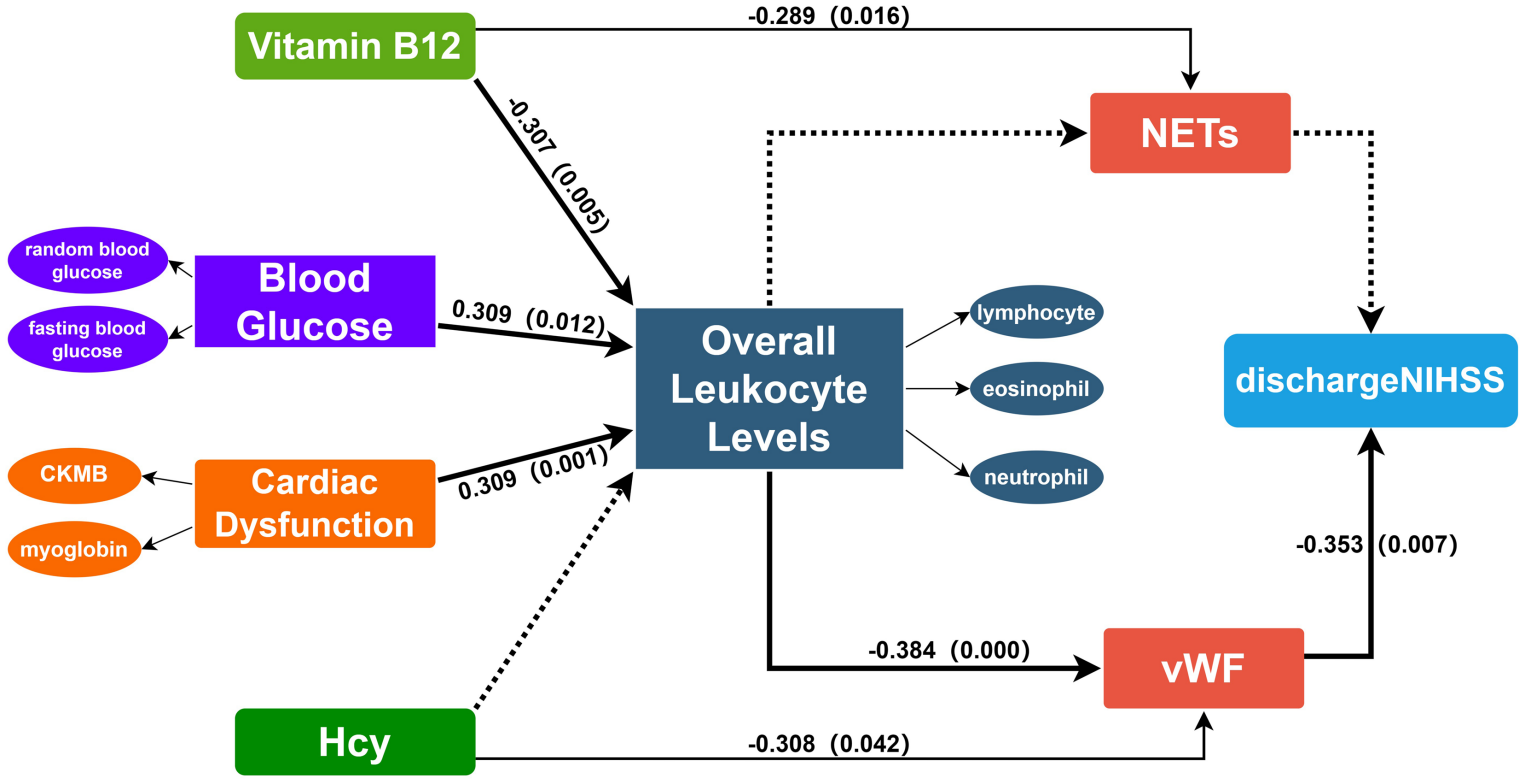
Final PLS-SEM model incorporating the following latent constructs: NETs, VWF, cardiac dysfunction, overall leukocyte levels, blood glucose, discharge NIHSS, vitamin B12. Overall leukocyte levels are effectively represented by neutrophils, lymphocytes and eosinophils. Blood glucose is represented by fasting blood glucose and random blood glucose. Cardiac dysfunction is represented by CKMB and Myoglobin. VWF and NETs are represented by the AOD measured in five different fields of view. PLS-SEM defines the relationships between latent variables and observed variables and among latent variables in the form of a path model. The directions of the explained paths are indicated by connecting arrows. Solid lines denote statistically significant paths, whereas dashed lines denote non-significant paths. Numbers on the arrows correspond to path coefficients, indicating the strength of each association. VWF, Von Willebrand factor; NETs, neutrophil extracellular traps; Hcy, Homocysteine; NIHSS, National Institutes of Health Stroke Scale.

The study included 61 patients with an average age of 60.49 years (Supplementary Table 1). The immunohistochemical staining positive areas are illustrated in Figure 1, showing a comparison of VWF and NETs staining in the same region across five images. In the initial model diagram, all variables were included. The model was iteratively adjusted by evaluating Cronbach’s alpha and outer loading coefficients. Final model retained significant paths and was simplified accordingly (Figure 2).

### 3.2 Model evaluation

Outer model evaluation validates the model showing strong construct validity. Overall leukocyte levels are effectively represented by neutrophils, lymphocytes and eosinophils, while other indicators appropriately reflect blood glucose and heart dysfunction (Supplementary Table 2). The Average Variance Extracted (AVE) and Cronbach’s alpha demonstrate good reliability and validity (Table 1).

**Table 1:** Reliability and validity of key variables.

	AVE			Cronbach’s alpha		
	Original sample (O)	M ± STDEV	*P*-value	Original sample (O)	M ± STDEV	*P*-value
Overall leukocyte levels	0.718	0.717 ± 0.041	<0.001	0.799	0.798 ± 0.041	<0.001
CARDIAC function	0.813	0.762 ± 0.129	<0.001	0.772	0.687 ± 0.209	<0.001
Blood glucose	0.774	0.739 ± 0.105	<0.001	0.713	0.707 ± 0.083	<0.001
Hcy	1.000	1.000 ± 0.000	n/a	1.000	1.000 ± 0.000	n/a
Vitamin B12	1.000	1.000 ± 0.000	n/a	1.000	1.000 ± 0.000	n/a
NETs	0.939	0.942 ± 0.016	<0.001	0.984	0.984 ± 0.004	<0.001
VWF	0.626	0.666 ± 0.069	<0.001	0.847	0.868 ± 0.040	<0.001
Discharge NIHSS	1.000	1.000 ± 0.000	n/a	1.000	1.000 ± 0.000	n/a

The Average Variance Extracted (AVE) values of all variables are greater than 0.5, the Cronbach’s alpha values are above 0.75, indicating good internal consistency and conforming to the requirements for convergent validity. AVE, Average Variance Extracted; M, mean; STDEV, Standard deviation; VWF, Von Willebrand Factor; NETs, Neutrophil Extracellular Traps, NIHSS, National Institutes of Health Stroke Scale.

All measurement model criteria were satisfied and confirmed its suitability for structural testing (Table 2). R square assess the explanatory power of the model for endogenous variables (25), indicating that vitamin B12, blood glucose, and heart dysfunction together accounted for 23.0% of the variance in overall leukocyte levels (*R* square = 0.230), while overall leukocyte levels and Hcy explained 24.4% of the variance in VWF (*R* square = 0.244). VWF and NETs in the thrombi accounted for 13.1% of the variance in NIHSS scores at discharge (*R* square = 0.131).

**Table 2:** Discriminant validity and model fit assessment.

HTMT original sample (O)	Hcy	NETs	VWF	Cardiac dysfunction	Overall leukocyte levels	Blood glucose	Discharge NIHSS
NETs	0.014						
VWF	0.339	0.450					
Cardiac dysfunction	0.046	0.144	0.313				
Overall leukocyte levels	0.108	0.322	0.461	0.386			
Blood glucose	0.125	0.110	0.090	0.094	0.309		
Discharge NIHSS	0.157	0.167	0.387	0.403	0.199	0.135	
Vitamin B12	0.276	0.336	0.213	0.055	0.215	0.325	0.018
HTMT sample mean (M)							
NETs	0.092						
VWF	0.314	0.464					
Cardiac dysfunction	0.161	0.202	0.423				
Overall leukocyte levels	0.125	0.327	0.467	0.456			
Blood glucose	0.157	0.183	0.180	0.278	0.335		
Discharge NIHSS	0.180	0.177	0.391	0.492	0.222	0.207	
Vitamin B12	0.277	0.342	0.242	0.175	0.230	0.335	0.105

R square	Original sample	M ± STDEV	SRMR
NETs	0.163	0.194 ± 0.080	0.069
VWF	0.244	0.270 ± 0.102	
Overall leukocyte levels	0.230	0.281 ± 0.091	
Discharge NIHSS	0.131	0.162 ± 0.084	

Discriminant validity is assessed by HTMT. All HTMT values being below 0.85, indicating good statistical differentiation among the variables (48). The evaluation of model fit showed an SRMR ≤ 0.090, indicating that the discrepancy between predicted and observed values was within acceptable limits (49). HTMT, Heterotrait-Monotrait ratio; STDEV, Standard deviation; M, mean; SRMR, Standardized Root Mean Square Residual; VWF, Von Willebrand Factor; NETs, Neutrophil Extracellular Traps; NIHSS, National Institutes of Health Stroke Scale.

### 3.3 Path coefficient

The path coefficient results indicate that overall leukocyte levels are significantly influenced by heart dysfunction, blood glucose, and vitamin B12 (Table 3). Vitamin B12 shows a negative effect (path coefficient = −0.307, *p* = 0.005). A one standard deviation decrease in vitamin B12 levels leads to a 0.307 unit increase in overall leukocyte levels. While heart dysfunction and blood glucose exhibit positive effects (path coefficient = 0.309, *p* = 0.001; path coefficient = 0.309, *p* = 0.012, respectively). Overall leukocyte levels have a significant negative impact on VWF (path coefficient = −0.384, *p* < 0.001), indicating that increased leukocyte levels correspond to a decrease in VWF content in thrombi. We also validated the impact of NETs and VWF on clinical symptom scores via the same approach. The path coefficients indicate that VWF negatively impacts NIHSS scores at discharge with a path coefficient of −0.353 (*p* = 0.007). A one standard deviation decrease in VWF content results in a 0.353 standard deviation increase in the NIHSS score at discharge, indicating worse early prognosis. Additionally, Hcy has a significant negative effect on VWF (path coefficient = −0.308, *p* = 0.046), and NETs are notably negatively influenced by vitamin B12 (path coefficient = −0.289, *p* = 0.016).

**Table 3:** Direct path coefficients in PLS-SEM model for risk factors, overall leukocyte levels and thrombus components.

Path coefficient	Original sample	M ± STDEV	T statistics	*p*-values
Hcy - > overall leukocyte levels	−0.045	−0.057 ± 0.074	0.613	0.540
Hcy - > VWF	−0.308	−0.281 ± 0.151	2.035	**0.042**
NETs - > discharge NIHSS	0.020	0.002 ± 0.138	0.142	0.887
Overall Leukocyte levels - > NETs	0.231	0.23 ± 0.131	1.769	0.077
Overall leukocyte levels - > VWF	−0.384	−0.392 ± 0.108	3.566	**<0.001**
Cardiac function - > overall leukocyte levels	0.309	0.330 ± 0.092	3.347	**0.001**
Glucose - > overall leukocyte levels	0.309	0.303 ± 0.122	2.525	**0.012**
VWF - > discharge NIHSS	−0.353	−0.372 ± 0.131	2.703	**0.007**
Vitamin B12 - > NETs	−0.289	−0.295 ± 0.12	2.401	**0.016**
Vitamin B12 - > overall leukocyte levels	−0.307	−0.311 ± 0.11	2.784	**0.005**

STDEV, standard deviation; M, mean; VWF, Von Willebrand Factor; NETs, neutrophil extracellular traps; Hcy, homocysteine, NIHSS, National Institutes of Health Stroke Scale. Bold values indicate statistically significant results at *p* < 0.05.

Subsequent analysis of indirect effects revealed statistically significant results for the effects of vitamin B12, blood glucose, and heart dysfunction on VWF through overall leukocyte levels (Table 4). An increase of one standard deviation in vitamin B12 leads to an indirect increase of 0.118 standard deviations in VWF through overall leukocyte levels. When combined changes in random and fasting blood glucose result in an overall increase of one standard deviation in blood glucose, VWF in thrombi decreases by 0.118 standard deviations through the mediation of leukocyte levels. For heart dysfunction, VWF decreases by 0.119 standard deviations. These findings demonstrate the overall effect of these exogenous variables on VWF content in thrombi when mediated by overall leukocyte levels.

**Table 4:** Specific indirect path coefficients in PLS-SEM model for risk factors, overall leukocyte levels and thrombus components.

Indirect effect	Original sample (O)	M ± STDEV	T statistics	*P*-values
Hcy - > overall leukocyte levels - > NETs	−0.010	−0.014 ± 0.022	0.468	0.639
Hcy - > overall leukocyte levels - > NETs - > discharge NIHSS	0.000	0.000 ± 0.004	0.054	0.957
Hcy - > overall leukocyte levels - > VWF	0.017	0.023 ± 0.032	0.552	0.581
Hcy - > overall leukocyte levels - > VWF - > discharge NIHSS	−0.006	−0.009 ± 0.014	0.452	0.651
Hcy - > VWF - > discharge NIHSS	0.109	0.103 ± 0.070	1.558	0.119
Overall leukocyte levels - > NETs - > discharge NIHSS	0.005	−0.001 ± 0.037	0.121	0.903
Overall leukocyte levels - > VWF - > discharge NIHSS	0.135	0.146 ± 0.066	2.040	**0.041**
Cardiac function - > overall leukocyte levels - > NETs	0.071	0.076 ± 0.050	1.423	0.155
Cardiac function - > overall leukocyte levels - > NETs - > discharge NIHSS	0.001	−0.001 ± 0.013	0.107	0.914
Cardiac function - > overall leukocyte levels - > VWF	−0.119	−0.131 ± 0.056	2.125	**0.034**
cardiac function - > overall leukocyte levels - > VWF - > discharge NIHSS	0.042	0.05 ± 0.029	1.429	0.153
Glusose - > overall leukocyte levels - > NETs	0.071	0.063 ± 0.043	1.663	0.096
Glusose - > overall leukocyte levels - > NETs - > discharge NIHSS	0.001	0.000 ± 0.011	0.129	0.897
Glusose - > overall leukocyte levels - > VWF	−0.118	−0.115 ± 0.051	2.313	**0.021**
Glusose - > overall leukocyte levels - > VWF - > discharge NIHSS	0.042	0.043 ± 0.026	1.633	0.103
Vitamin B12 - > NETs - > discharge NIHSS	−0.006	0.000 ± 0.044	0.129	0.898
Vitamin B12 - > overall leukocyte levels - > NETs	−0.071	−0.071 ± 0.050	1.419	0.156
Vitamin B12 - > overall leukocyte levels - > NETs - > discharge NIHSS	−0.001	0.000 ± 0.013	0.111	0.912
Vitamin B12 - > overall leukocyte levels - > VWF	0.118	0.123 ± 0.057	2.067	**0.039**
Vitamin B12 - > overall leukocyte levels - > VWF - > discharge NIHSS	−0.042	−0.046 ± 0.028	1.467	0.142

STDEV, Standard deviation; M, mean; VWF, Von Willebrand Factor; NETs, Neutrophil Extracellular Traps; Hcy, Homocysteine, NIHSS, National Institutes of Health Stroke Scale. Bold values indicate statistically significant results at *p* < 0.05.

### 3.4 Impact of confounding variables

To further assess the influence of potential confounders on model robustness, participants were stratified by several factors including chronic comorbidities, smoking and alcohol drinking. No significant differences were observed between sexes, nor did histories of hypertension, cerebrovascular disease or smoking. In the subgroup with a history of diabetes, only blood glucose differed significantly (Supplementary Table 3). Age-stratified analyses revealed increases in R square values across endogenous variables, indicating that over 60 years of age enhances the explanatory power of the PLS-SEM framework. In the drinker and normolipidemic subgroups, the model’s ability to predict discharge NIHSS scores also improved (Table 5). In normolipidemic subgroup, the influence of leukocyte count on VWF production was strengthened, and the mediated effects of blood glucose, cardiac dysfunction, and vitamin B12 on VWF via leukocytes were all amplified. The path coefficient show that overall leukocyte levels exert an even stronger inverse effect on VWF than in the full sample (path coefficient = −0.419, *p* < 0.001 in normolipidemic subgroup, path coefficient = −0.534, p < 0.001 in drinker subgroup). The mediated effects of blood glucose, cardiac dysfunction and vitamin B12 on VWF via leukocytes are all amplified in normolipidemic subgroup (Supplementary Table 4). These findings suggest that under normal lipid conditions, inflammatory mechanisms more effectively attenuate VWF synthesis.

**Table 5:** R square across groups stratified by different confounding variables.

R square	Age≥ 60		Age≤60		Normolipidemic		Drinker	
	Original sample	M ± STDEV	Original sample	M ± STDEV	Original sample	M ± STDEV	Original sample	M ± STDEV
NETs	0.190	0.247 ± 0.119	0.154	0.234 ± 0.143	0.156	0.189 ± 0.083	0.166	0.239 ± 0.126
Overall leukocyte levels	0.226	0.332 ± 0.125	0.307	0.404 ± 0.125	0.212	0.269 ± 0.092	0.120	0.333 ± 0.149
Discharge NIHSS	0.093	0.156 ± 0.113	0.241	0.298 ± 0.133	0.145	0.184 ± 0.094	0.362	0.395 ± 0.118
VWF	0.306	0.355 ± 0.145	0.176	0.249 ± 0.137	0.212	0.246 ± 0.103	0.308	0.359 ± 0.121

STDEV, Standard deviation; M, mean; VWF, Von Willebrand Factor; NETs, Neutrophil Extracellular Traps; NIHSS, National Institutes of Health Stroke Scale.

## 4 Discussion

Research on thrombi components of AIS patients provides insight into the pathological changes during the stroke process. VWF and NETs are significant contributors in immune-inflammatory responses during disease process and also serve as key therapeutic targets. Our study revealed a correlation between VWF levels in thrombi and early clinical outcomes, and it identified that cerebral risk factors blood glucose, heart dysfunction, and vitamin B12 influence VWF content in thrombi through overall leukocyte levels. This finding demonstrates that inflammation represented by leukocytes acts as a bridge connecting clinical laboratory results with pathological findings. And it is worth noting that age over 60 is a key factor in enhancing the model’s explanatory power.

In this study, applying a structural equation model, we identified that lower VWF in thrombi was significantly associated with higher NIHSS scores at discharge which indicates more severe clinical symptoms. As previous studies have shown, VWF level is associated with increased symptom severity and poorer clinical prognosis in AIS patients (26, 27). Leukocytes promote the activation and aggregation of platelets, also secrete procoagulant substances that trigger coagulation cascade during stroke (28). Following thromboembolic events, PSGL-1 receptors and integrins on the leukocyte surface interact with the A1 domain of VWF, facilitating leukocyte migration to injured areas (29). In parallel, VWF-platelet complexes can modulate vascular permeability, reducing barriers to leukocyte migration. This process accelerates leukocyte rolling, adhesion, and extravasation, thereby exacerbating the inflammatory response (30). Intense inflammation further triggers the release of VWF from Weibel-Palade bodies in endothelial cells, initiating downstream cascades. Our study identified a significant negative association between peripheral overall leukocyte levels in the early phase of AIS and VWF content in thrombi, suggesting that increased inflammation is accompanied by a reduction in VWF within thrombi.

Inflammatory responses are linked to various factors. Evidence has shown that blood glucose combined with leukocyte count predicts in-hospital mortality in AIS patients (31). In diabetic patients, peripheral leukocyte count and VWF show an increasing tendency (32), leading to the increase in thrombotic risk for diabetic patients (33). Using PLS-SEM, we revealed that when overall leukocyte levels act as a mediator, blood glucose exerts a significant negative indirect effect on VWF, leading to higher NIHSS scores at discharge. Previous studies have identified that hyperglycemia suppresses microRNA-24 and regulates VWF secretion (34). Vitro experiments demonstrate that high blood glucose levels reduce nitric oxide and induce endothelial hypoxia via fibrinogen, modifying the levels of NF-I and NF-Y (repressors of the VWF promoter). This also facilitates the binding of transcription factor YY1 to the VWF promoter, thereby modulating VWF secretion (35, 36). There are also studies found leukocyte release human neutrophil peptides to inhibit proteolytic cleavage of VWF by ADAMTS13 in a concentration-dependent manner (37).

Clinical data strongly suggest that vitamin B12 deficiency is a risk factor influencing the severity and outcome of stroke (38, 39). Some researchers suggest that vitamin B12 supports gut-brain homeostasis during stroke by balancing gut immunity and enhancing microbial metabolite utilization (40). Our study found that vitamin B12 deficiency raises overall leukocyte levels, leading to a reduction in VWF content in thrombi, which is closely linked to poor early prognosis. Previous studies found that vitamin B12 shows an anti-inflammatory and neuroprotective action through methyl-dependent epigenetic mechanisms (41). Mice with vitamin B12 deficiency exhibited increased inflammatory marker levels such as IL-6, accompanied by leukocyte activation (42). Current evidence indicates that vitamin B12 deficiency often co-exists with thrombotic thrombocytopenic purpura, a lethal micro-thrombotic disorder caused by severe ADAMTS13 deficiency and abnormal VWF accumulation (43). Some researchers suggest that vitamin B12 might facilitate or even trigger abnormal VWF deposition in thrombotic disorders (44). We also found a direct and significant negative effect of vitamin B12 on NETs within thrombi. As a scaffold for cells and various coagulation factors, NETs induce oxidative stress, increase the expression of coagulation factors, and lead to proinflammatory reactions (45). Current research suggests that vitamin B12 deficiency may influence the production of superoxide and peroxynitrite in circulating leucocytes and resident cells of the vessel wall (46). Moreover, the direct scavenging of reactive oxygen species by vitamin B12 may impede NETs formation and the associated oxidative stress (47). This study reveals a potential link between vitamin B12 and thrombi component NETs, providing new insights into exploring related pathological mechanisms and offering clinicians a new and practically quantifiable peripheral biomarker for targeted NETs therapy.

## 5 Implications

Previous studies on thrombi formation in AIS are mostly limited to laboratory research or pathological analysis. This is the first comprehensive exploration of the relationships between clinical laboratory markers, clinical symptoms, and thrombi components. We demonstrated that inflammatory response serves as a critical mediator linking thrombi VWF with AIS risk factors, including blood glucose, vitamin B12, and the degree of heart dysfunction. Additionally, we confirmed a significant association between VWF content in thrombi and early clinical prognosis. This study integrates previous independent research through structural equation modeling, bridging basic medical research with clinical practice, and provides new insights and methods for future investigations into pathogenic mechanisms.

The content of NETs and VWF within thrombi is linked to disease prognosis. Clinically, direct assessment of thrombi components is challenging without thrombectomy, and even post-thrombectomy component analysis may not be promptly available. This study found the significant associations between thrombi components and clinical markers. These findings suggest a new approach: assessing these commonly available clinical markers could help clinicians infer thrombi composition from peripheral blood tests, facilitating targeted therapy and early disease prevention. These findings also provide valuable insights into the pathological mechanisms underlying thromboinflammation in AIS, reinforcing the importance of clinical assessment and guiding the development of early targeted interventions.

Our study has certain limitations. The sample size was relatively small, and thus further large-scale studies are required to validate the accuracy of the model. The temporal evolution of leukocyte levels, VWF, and NETs during stroke progression are not examined. Additionally, the depth of basic research and targeted therapeutic approaches was constrained by available techniques. The precise mechanisms by which leukocyte activity influences thrombi formation remain to be elucidated. Moreover, characterizing how VWF, NETs, and other key indicators evolve throughout stroke progression represents an important avenue for future research, as it could deepen our understanding of the relationship between the pathophysiology of cerebral infarctio n and clinical biomarkers. Future studies should involve larger sample sizes and more comprehensive analyses to further verify and refine the findings.

## 6 Conclusion

The PLS-SEM data model revealed a close association between decreased VWF content in thrombi and poor early clinical outcomes. Among various cerebral risk factors, the inflammatory response represented by overall leukocyte levels mediates the impact of blood glucose, heart dysfunction, and vitamin B12 on the thrombi component VWF. Elevated blood glucose and heart dysfunction lead to a reduction of VWF through rising overall leukocyte levels. Vitamin B12 deficiency results in higher overall leukocyte levels, indirectly reducing VWF content in thrombi. Additionally, elevated Hcy levels are significantly associated with decreased VWF, while higher vitamin B12 levels are linked to a reduction in NETs within thrombi.

## Data Availability

The raw data supporting the conclusions of this article will be made available by the authors, without undue reservation.
