# Supplementary material for: Overall leukocyte levels link risk factors to Von Willebrand factor and Neutrophil Extracellular Traps in stroke thrombi: a Structural Equation Modeling analysis
Source: Front Neurol. 2025 Jun 23;16:1515596. doi: 10.3389/fneur.2025.1515596 (PMC12229865; doi:10.3389/fneur.2025.1515596)
Supplement: Supplementary file 1 [file Table_1.docx]

Supplementary Table 1 descriptive statistics

|  | Median | 1st Quartile | 3rd Quartile | Mean |
| --- | --- | --- | --- | --- |
| VWF1 | 213.9883 | 209.0192 | 220.0757 | 209.7403 |
| VWF2 | 212.6823 | 208.6019 | 217.6153 | 213.0992 |
| VWF3 | 213.2958 | 209.0616 | 218.0105 | 213.1928 |
| VWF4 | 213.7968 | 207.7693 | 219.3410 | 212.9090 |
| VWF5 | 212.9606 | 207.5771 | 218.9379 | 209.3737 |
| NETs1 | 197.4993 | 164.0134 | 214.8995 | 186.6250 |
| NETs2 | 195.0016 | 155.1091 | 214.9648 | 186.9407 |
| NETs3 | 207.1484 | 154.0584 | 215.9822 | 186.9731 |
| NETs4 | 188.7717 | 154.1926 | 213.4885 | 185.3626 |
| NETs5 | 206.7468 | 152.7036 | 215.4678 | 184.3245 |
| HbA1c | 6.1 | 5.5 | 7.0 | 6.6200 |
| Fasting blood glucose | 6.73 | 5.6 | 8.55 | 7.3535 |
| Random blood glucose | 7.44 | 6.57 | 9.0 | 8.3309 |
| Vitamin B12 | 222 | 155 | 333.7 | 262.7098 |
| Hcy | 13.3 | 11 | 17 | 18.7449 |
| CKMB | 1.32 | 0.71 | 1.82 | 1.7063 |
| BNP | 184 | 78.7 | 512 | 640.69 |
| Myoglobin | 61.6 | 36.5 | 107.1993 | 98.4662 |
| White Blood Cells Count | 9.57 | 8.26 | 11.45 | 10.0308 |
| Neutrophil Count | 7.1484 | 6.0298 | 9.765 | 7.9837 |
| Lymphocyte Count | 1.288 | 0.987 | 1.9467 | 1.4476 |
| Monocyte Count | 0.4956 | 0.3711 | 0.658 | 0.5444 |
| Eosinophil Count | 0.02 | 0.0001 | 0.06 | 0.0451 |
| Calcium | 2.14 | 2.03 | 2.25 | 2.1522 |
| Potassium | 3.96 | 3.77 | 4.17 | 3.9636 |
| Sodium | 139.2 | 137 | 140.5 | 139.21 |
| Chloride | 103.8 | 101.6 | 106 | 103.83 |
| Urea | 5.5 | 4.6 | 6.94 | 6.1857 |
| Creatinine | 64.8 | 55.7 | 73.1 | 66.6098 |
| Uric Acid | 309 | 254 | 364 | 306.8239 |
| LDL | 2.61 | 2.12 | 3.11 | 2.7022 |
| HDL | 1.01 | 0.84 | 1.18 | 1.0457 |
| Triglycerides | 1.62 | 1.15 | 2.03 | 1.7849 |
| Cholesterol | 4.44 | 3.56 | 4.78 | 4.3601 |
| Total Bilirubin | 14.5 | 11.3 | 18.8 | 15.66 |
| Direct Bilirubin | 3.4 | 2.8 | 4.3 | 3.85 |
| Indirect Bilirubin | 10.6 | 7.4 | 14.5 | 11.8 |
| Total Protein | 62.1 | 57.2 | 66.4 | 61.9163 |
| Albumin | 37.3 | 34.9 | 39.2 | 41.72 |
| AST | 21.7 | 16.7 | 27.4 | 23.9737 |
| ALT | 18.3 | 12.8 | 25.9 | 23.2131 |
| Glutamyl Transferase | 30.8 | 21.1 | 47.6 | 38.07541 |
| Alkaline Phosphatase | 72.6 | 59.4 | 86.2 | 75.54918 |
| Cholinesterase | 7090 | 6540 | 8152 | 7454.656 |
| Admission NIHSS | 15 | 11 | 19 | 17.6 |
| Discharge NIHSS | 6 | 2 | 21 | 12.1 |

VWF: von Willebrand Factor; NETs: Neutrophil Extracellular Traps; HbA1c: Hemoglobin A1c; Hcy: Homocysteine; BNP: B-type Natriuretic Peptide; CKMB: Creatine Kinase-MB; LDL: Low-Density Lipoprotein; HDL: High-Density Lipoprotein; AST: Aspartate Aminotransferase; ALT: Alanine Aminotransferase; NIHSS: National Institutes of Health Stroke Scale.

Supplementary Table 2 Outer Loadings

|  | Original sample | mean | Standard deviation | T statistics | P values |
| --- | --- | --- | --- | --- | --- |
| Box-Cox conversed NETs1ave <- NETs | 0.957 | 0.957 | 0.015 | 61.792 | 0.000 |
| Box-Cox conversed NETs2ave <- NETs | 0.980 | 0.981 | 0.006 | 174.592 | 0.000 |
| Box-Cox conversed NETs3ave <- NETs | 0.981 | 0.981 | 0.007 | 148.837 | 0.000 |
| Box-Cox conversed NETs4ave <- NETs | 0.981 | 0.981 | 0.004 | 218.477 | 0.000 |
| Box-Cox conversed NETs5ave <- NETs | 0.946 | 0.950 | 0.029 | 32.237 | 0.000 |
| Box-Cox conversed VWF1ave <- VWF | 0.750 | 0.795 | 0.078 | 9.604 | 0.000 |
| Box-Cox conversed VWF2ave <- VWF | 0.800 | 0.796 | 0.071 | 11.316 | 0.000 |
| Box-Cox conversed VWF3ave <- VWF | 0.839 | 0.833 | 0.049 | 17.194 | 0.000 |
| Box-Cox conversed VWF4ave <- VWF | 0.886 | 0.887 | 0.037 | 24.110 | 0.000 |
| Box-Cox conversed VWF5ave <- VWF | 0.649 | 0.720 | 0.143 | 4.538 | 0.000 |
| Fasting Blood Glucose <- Glucose | 0.845 | 0.797 | 0.203 | 4.169 | 0.000 |
| Random blood glucose <- Glucose | 0.913 | 0.881 | 0.163 | 5.594 | 0.000 |
| CKMB <- cardiac function | 0.923 | 0.902 | 0.124 | 7.424 | 0.000 |
| Myoglobin <- cardiac function | 0.880 | 0.807 | 0.210 | 4.196 | 0.000 |
| Neutrophil <- Overall Leukocyte Levels | 0.912 | 0.908 | 0.031 | 28.952 | 0.000 |
| Eosinophil <- Overall Leukocyte Levels | 0.739 | 0.740 | 0.069 | 10.702 | 0.000 |
| lymphocyte <- Overall Leukocyte Levels | 0.881 | 0.879 | 0.045 | 19.742 | 0.000 |
| Vitamin B12 <- vitamin B12 | 1.000 | 1.000 | 0.000 | n/a | n/a |
| Hcy <- Hcy | 1.000 | 1.000 | 0.000 | n/a | n/a |

Outer loadings of all observed variables are above 0.70, indicating strong construct validity of model. VWF: von Willebrand Factor; NETs: Neutrophil Extracellular Traps; CKMB: Creatine Kinase-MB; Hcy: Homocysteine.

Supplementary Table 3 P values calculated by Mann–Whitney U test

| P value | Gender | Age | Hypertension | Hyperlipidemia | Diabetes mellitus | cerebrovascular disease | smoker | drinker |
| --- | --- | --- | --- | --- | --- | --- | --- | --- |
| NETs1 | 0.329 | 0.452 | 0.208 | 0.086 | 0.241 | 0.566 | 0.475 | 0.794 |
| NETs2 | 0.115 | 0.563 | 0.318 | 0.160 | 0.592 | 0.646 | 0.274 | 0.442 |
| NETs3 | 0.268 | 0.839 | 0.188 | 0.716 | 0.947 | 0.646 | 0.600 | 0.442 |
| NETs4 | 0.378 | 0.553 | 0.208 | 0.175 | 0.675 | 0.555 | 0.466 | 0.601 |
| NETs5 | 0.133 | 0.707 | 0.156 | 0.146 | 0.675 | 0.294 | 0.091 | 0.223 |
| VWF1 | 0.293 | 0.582 | 0.325 | 0.095 | 0.492 | 0.623 | 0.274 | 0.070 |
| VWF2 | 0.277 | 0.219 | 0.851 | 0.066 | 0.947 | 0.051 | 0.096 | **0.019** |
| VWF3 | 0.245 | 0.885 | 0.839 | 0.069 | 0.524 | 0.670 | 0.314 | **0.011** |
| VWF4 | 0.223 | 0.919 | 0.908 | **0.037** | 0.893 | 0.555 | 0.221 | 0.146 |
| VWF5 | 0.431 | 0.919 | 0.664 | **0.021** | 0.713 | 0.309 | 0.166 | 0.065 |
| Discharge NIHSS | 0.604 | 0.771 | 0.913 | 0.567 | 0.438 | 0.219 | 0.291 | 0.948 |
| Fasting blood glucose | 0.881 | 0.739 | 0.592 | **0.042** | **0.001** | 0.062 | 0.418 | 0.127 |
| Random blood glucose | 0.209 | 0.241 | 0.529 | 0.090 | **0.008** | 0.305 | 0.112 | 0.320 |
| Vitamin B12 | 0.399 | **0.024** | 0.891 | 0.287 | 0.119 | 0.393 | 0.212 | 0.380 |
| Hcy | 0.659 | 0.925 | 0.654 | 0.865 | 0.860 | 0.075 | 0.120 | 0.931 |
| CKMB | 0.925 | 0.064 | 0.418 | 0.837 | 0.604 | 0.367 | 0.765 | 0.760 |
| Myoglobin | 0.978 | **0.003** | 1.000 | 0.561 | 0.639 | 0.112 | 0.347 | 0.642 |
| Neutrophil Count | 0.910 | 0.213 | 0.332 | 0.498 | 0.987 | 0.658 | 0.873 | 0.360 |
| Lymphocyte Count | 0.499 | 0.874 | 0.643 | 0.545 | 0.675 | 0.909 | 0.126 | 0.212 |
| Eosinophil Count | 0.696 | 0.28 | 0.336 | 0.863 | 0.481 | 0.583 | 0.756 | 0.591 |

VWF: von Willebrand Factor; NETs: Neutrophil Extracellular Traps; CKMB: Creatine Kinase-MB; Hcy: Homocysteine

Supplementary Table 4 Direct Path Coefficients in normolipidemic subgroup and drinker subgroup

| Path Coefficient in normolipidemic subgroup | Original sample (O) | M±STDEV | T statistics | P values |
| --- | --- | --- | --- | --- |
| cardiac function -> Overall Leukocyte Levels | 0.303 | 0.323±0.126 | 2.404 | **0.016** |
| glusose -> Overall Leukocyte Levels | 0.276 | 0.273±0.129 | 2.145 | **0.032** |
| Hcy -> Overall Leukocyte Levels | -0.116 | -0.122±0.089 | 1.312 | 0.190 |
| Hcy -> vWF | -0.193 | -0.169±0.147 | 1.313 | 0.189 |
| NETs -> discharge NIHSS | 0.017 | -0.002±0.148 | 0.117 | 0.907 |
| Overall Leukocyte Levels -> NETs | 0.234 | 0.233±0.134 | 1.750 | 0.080 |
| Overall Leukocyte Levels -> vWF | -0.419 | -0.431±0.112 | 3.725 | **0.000** |
| vitaminB12 -> NETs | -0.278 | -0.283±0.126 | 2.212 | **0.027** |
| vitaminB12 -> Overall Leukocyte Levels | -0.340 | -0.338±0.116 | 2.926 | **0.003** |
|  |  |  |  |  |
| Path Coefficient in drinker subgroup | Original sample (O) | M±STDEV | T statistics | P values |
| cardiac function -> Overall Leukocyte Levels | 0.077 | 0.115±0.380 | 0.202 | 0.840 |
| glusose -> Overall Leukocyte Levels | 0.322 | 0.312±0.328 | 0.980 | 0.327 |
| Hcy -> Overall Leukocyte Levels | -0.059 | -0.085±0.238 | 0.249 | 0.803 |
| Hcy -> vWF | 0.135 | 0.135±0.149 | 0.905 | 0.365 |
| NETs -> discharge NIHSS | 0.156 | 0.103±0.195 | 0.798 | 0.425 |
| Overall Leukocyte Levels -> NETs | 0.311 | 0.311±0.207 | 1.506 | 0.132 |
| Overall Leukocyte Levels -> vWF | -0.534 | -0.558±0.128 | 4.174 | **0.000** |
| vitaminB12 -> NETs | -0.004 | -0.015±0.202 | 0.021 | 0.984 |
| vitaminB12 -> Overall Leukocyte Levels | -0.134 | -0.136±0.232 | 0.579 | 0.562 |
| vWF -> discharge NIHSS | -0.470 | -0.527±0.143 | 3.290 | **0.001** |

STDEV: Standard deviation. M: mean; VWF: Von Willebrand Factor. NETs: Neutrophil extracellular traps. Hcy: Homocysteine, NIHSS: National Institutes of Health Stroke Scale.
